# Metformin attenuates the production and proliferative effects of prolactin induced by medroxyprogesterone acetate during fertility-sparing treatment for endometrial cancer

**DOI:** 10.1186/s12885-022-09858-w

**Published:** 2022-07-11

**Authors:** Wenjing Gu, Akira Mitsuhashi, Tatsuya Kobayashi, Makio Shozu

**Affiliations:** 1 Department of Reproductive Medicine, Graduate School of Medicine, Chiba University, Chiba, Japan; 2 Department of Obstetrics and Gynecology, School of Medicine, Dokkyo Medical University, Tochigi, Japan

**Keywords:** Prolactin, Metformin, Medroxyprogesterone acetate, Fertility preservation, Endometrial neoplasms

## Abstract

**Background:**

Progestin is used for fertility-sparing treatment in cases of endometrial cancer (EC). Progestin can induce hyperprolactinemia by increasing pituitary secretion and endometrial decidualization. However, progestin induces prolactin (PRL) secretion, which stimulates cell proliferation and deleteriously affects treatment. To date, the detrimental effect of PRL, the secretion of which is induced by medroxyprogesterone acetate (MPA) during fertility-sparing treatment, has not yet been fully elucidated. Therefore, we aimed to assess the effects of PRL on EC cells during combined treatment with progestin and metformin.

**Methods:**

In total, 71 patients with EC/endometrial atypical hyperplasia who underwent fertility-sparing treatment at our institution from 2009–2019 were enrolled. Serum PRL levels were determined using enzyme immunoassays; mRNA levels in endometrial tissues were determined using quantitative reverse-transcription PCR. To evaluate MPA-induced decidualization, cancer-associated stromal cells were enzymatically released from surgically removed specimens of six patients with EC. To examine PRL-induced cell proliferation, the EC cell lines Ishikawa, HEC1B, and HEC265 were used. In vitro cell proliferation was evaluated using the WST assay; protein levels of signaling molecules were determined using western blotting.

**Results:**

MPA administration significantly increased serum PRL levels at 3 and 6 months and upregulated *IGFBP-1* and *PRL* mRNA expression in tissues at 3 months of fertility-sparing treatment. Metformin significantly reduced MPA-induced *IGFBP-1* and *PRL* mRNA expression during fertility-sparing treatment and significantly inhibited the upregulation of *IGFBP-1* and *PRL* mRNA and PRL levels due to decidualization induced by MPA and cAMP treatment in primary cultured EC stromal cells. In vitro, PRL increased cell proliferation and ERK1/2 phosphorylation levels, whereas metformin attenuated these increases.

**Conclusions:**

MPA upregulated PRL levels in serum and endometrial tissues during fertility-sparing treatment. Metformin co-administration reduced PRL production and attenuated PRL-induced cell-proliferation activity. This study may provide valuable insights on the application of metformin to improve the outcomes of fertility-sparing treatment.

**Supplementary Information:**

The online version contains supplementary material available at 10.1186/s12885-022-09858-w.

## Background

Prolactin (PRL) is a hormone that is mainly secreted by the pituitary gland; it plays a role in several processes, including development of the mammary gland, lactation, implantation and pregnancy, angiogenesis, and regulation of immune function. PRL is also secreted by extrapituitary sites, including the mammary gland and endometrium, and acts locally as a growth factor [1, 2]. Increasing evidence suggests the stimulatory effects of PRL on several cancers, such as lymphoid, mammary, colon, hepatocellular, prostate, ovarian, and endometrial carcinomas [3–8]. Human PRL (hPRL) expression has been reported to be upregulated in endometrial cancer (EC) and is associated with poor survival outcomes [4]. Autocrine hPRL expression in EC cells promotes their proliferation, migration, and invasion [9].

Uterine cancer is the fourth most common cancer among women in the USA, and its incidence has increased by about 1% each year since the mid-2000s [10]. Moreover, the number of patients with endometrial atypical hyperplasia (EAH) and EC who desire to preserve their fertility is increasing. Progestin therapy is a popular treatment option for preserving the fertility of these patients [11, 12]. However, the results of three meta-analyses revealed high rates of both remission and relapse [13–15]. Progestin induces hyperprolactinemia, similar to anti-dopamine antagonists [16, 17], and increases PRL levels via decidualization of the endometrium [18]. However, levels of PRL during progestin administration for fertility-sparing treatment and the consequent effects on treatment are not well-understood.

Metformin, a biguanide, is commonly prescribed for the treatment of type 2 diabetes and has been attracting increasing attention in the field of cancer research. Population-based studies suggest that metformin decreases the incidence of cancer and cancer-related mortality in patients with diabetes [19, 20]. Metformin has also been associated with improved recurrence-free and overall survival in patients with EC with diabetes [21, 22]. We previously reported the efficacy of combining metformin and progestin to improve the long-term oncological outcomes of these patients [23, 24].

In this study, we aimed to investigate PRL levels before and during medroxyprogesterone 17-acetate (MPA; a progestin) treatment. We also aimed to evaluate the effects of metformin on the production of and interaction with PRL during MPA treatment in patients with EAH or EC. The findings of this study might provide valuable insights into the use of metformin to improve the outcomes of fertility-sparing treatment.

## Methods

### Patients and clinical samples

From 2009 to 2019, 86 patients with EAH and EC were treated by administering MPA, with or without metformin, as fertility-sparing treatment at our institution. The eligibility criteria for the administration of MPA and metformin as a fertility-sparing treatment have been described previously [23]. We only included patients who were not on any PRL-increasing medications and had no other medical conditions that could increase PRL levels. Among these patients, 71 were included in this study because we could obtain the relevant laboratory data related to serum PRL levels before and during MPA treatment. Patient blood samples were collected at the outpatient clinic between 9–11 am. PRL was measured using a chemiluminescent immunoassay with the ARCHITECT i2000SR and 4000SR Immunoassay Analyzer (Abott Diagnostics, USA). The patient characteristics are listed in Table 1.

**Table 1:** Patients’ characteristic

	Median (range)	N (%)
Age (year)	35 (25–45)	
Histology Endometrioid carcinoma, grade 1		43 (60.6)
EAH		28 (39.4)
Treatment MPA + Metformin		51 (71.8)
MPA alone		20 (28.2)
BMI (kg/m^2^)	30.4 (15.4–50.3)	
BMI (kg/m^2^) ≥ 25		47 (66.2)
HOMA-IR	4.0 (0.1–20.7)	
HOMA-IR ≥ 2.5		41 (57.7)
PCOS		55 (77.5)

*EAH* endometrial atypical hyperplasia, *MPA* medroxyprogesterone acetate, *BMI* body mass index, *HOMA-R* homeostasis model assessment of insulin resistance, *PCOS* polycystic ovarian syndrome

Paired EC tissues were obtained from patients with EC who underwent fertility-sparing treatment with MPA and metformin (*n* = 11) or with MPA alone (*n* = 5) before and after MPA administration. Tissue specimens were obtained via endometrial curettage at the time of initial diagnosis (before treatment) and 3 months after the start of MPA treatment. Specimens were snap-frozen in liquid nitrogen and stored at − 80 °C for subsequent analyses.

Additionally, we obtained tissues from patients with grade 1, stage IA endometrioid carcinoma who had undergone hysterectomy and had not received progestin before surgery (*n* = 6) to collect primary EC culture cells. This study was approved by the Institutional Review Board of Chiba University (IRB No. 3837). Before participation, written informed consent for use of specimens was obtained from the six patients whose tissue was newly collected. The opt-out approach was applied to obtain consent to extract patient data from digital medical records and for the use of stored samples.

### Reagents

Antibodies against phospho-p44/42 mitogen-activated protein kinase (MAPK; phospho-extracellular signal-regulated kinase [ERK] 1/2; Thr202/Tyr204; 1:2,000 dilution; catalog #4370S), p44/42 MAPK (ERK 1/2; 1:1,000 dilution; catalog #4695S), phospho-ribosomal protein S6 (rpS6) (Thr389; 1:1,000 dilution; catalog #2215S), rpS6 (1:1,000 dilution; #2217S), and β-actin (1:5,000 dilution; catalog #4970), and anti-rabbit IgG horseradish peroxidase (HRP)-linked secondary antibody (catalog #NA934V) were purchased from Cell Signaling Technology (Danvers, MA, USA). Metformin (catalog #D150959-5G), deoxyribonuclease I (from bovine pancreas, catalog #DN25-1G), collagenase (from *Clostridium histolyticum*, catalog #C0130-5G), MPA (catalog #M1629-1G), and E_2_ (β-estradiol, catalog #250,155-1G) were obtained from Sigma-Aldrich (St. Louis, MO, USA); 8-bromoadenosine-3ʹ,5ʹ-cyclic monophosphate sodium salt hydrate (cyclic adenosine monophosphate [cAMP] analog; catalog #05,450–02) was obtained from Nacalai Tesque (Tokyo, Japan).

### Cell lines and culture

Three type 1 EC model cell lines (Ishikawa, HEC265, and HEC1B) were cultured in Dulbecco’s modified Eagle’s medium (DMEM; Gibco, Thermo Fisher Scientific, Waltham, MA, USA) containing 4.5 g/L glucose, 5% fetal bovine serum (FBS; Sigma-Aldrich), 100 U/mL penicillin, and 100 μg/mL kanamycin sulfate at 37 °C and 5% CO_2_. HEC265 and HEC1B cell lines were purchased from the Japanese Collection of Research Bioresources Cell Bank (Osaka, Japan). The Ishikawa cell line was generously provided by Dr. Nishida (Tsukuba University, Japan).

### Cell proliferation assay (WST assay)

Cells were seeded in 96-well plates at a density of 3,500 cells/well in DMEM containing 5% FBS for 24 h. To explore the effect of PRL, recombinant hPRL was added at a final concentration of 0–1 μg/mL. Additionally, to explore the inhibitory effect of metformin on PRL, we examined the effects of the combination of PRL and metformin (1 mM). Cells were cultured for an additional 94 h and the absorbance was measured at 570 nm using an automated microplate reader (Infinite 200; Tecan, Männedorf, Switzerland).

### Western blot assay

Total protein was extracted from EC cells (HEC265 and HEC1B) 6 h after the addition of PRL with or without metformin after the cells reached 80% confluence using cOmplete Lysis-M buffer (Roche Applied Science, Tokyo, Japan) containing Halt Phosphatase Inhibitor Cocktail (Thermo Fisher Scientific) and quantified using the Pierce BCA Protein Assay Kit (Thermo Fisher Scientific). The obtained protein (20 μg) was subjected to 10% sodium dodecyl sulfate–polyacrylamide gel electrophoresis and electrotransferred onto polyvinylidene fluoride membranes (GE Healthcare, Chicago, IL, USA). Next, the membranes were blocked with 5% non-fat milk during a 1-h incubation at 25 ℃.

The secondary antibodies (enhanced chemiluminescence HRP-conjugated anti-rabbit IgG and anti-mouse IgG; GE Healthcare) were incubated with the membranes at room temperature for 60 min. Signals were detected using Amersham ECL Prime Western Blotting Detection Reagent (GE Healthcare). Signal intensity was quantified using a densitometer (CS Analyzer version 3.0; ATTO, Tokyo, Japan) and normalized to β-actin levels.

### Reverse transcription PCR (RT-PCR) analysis

Total RNA was extracted from cells and tissues using the RNeasy Mini Kit (Qiagen, Hilden, Germany). RNA was reverse transcribed into cDNA using the SuperScript VILO cDNA Synthesis Kit (Thermo Fisher Scientific) according to the manufacturer’s protocol. Finally, the expression of PRL, insulin-like growth factor-binding protein 1 (IGFBP-1), progesterone receptor (PgR), and estrogen receptor-α (ERα) was determined using specific quantitative primers and SYBR Green PCR Master Mix (Thermo Fisher Scientific) with cDNA as the template and TUBB (β-tubulin) as the endogenous control. The quantitative primer sequences are as follows:

*PRL*, 5ʹ-CATATTGCGATCCTGGAATGAGC-3ʹ (forward) and 5ʹ-TCCTCAATCTCTACAGCTTTGGA-3ʹ (reverse); *IGFBP-1*, 5ʹ-TCCTTTGGGACGCCATCAGTAC-3ʹ (forward) and 5ʹ-GATGTCTCCTGTGCCTTGGCTA-3ʹ (reverse); *PgR*, 5ʹ-GTCGCCTTAGAAAGTGCTGTCAG-3ʹ (forward) and 5ʹ-GCTTGGCTTTCATTTGGAACGCC-3ʹ (reverse); *ERα*, 5ʹ-GCTTACTGACCAACCTGGCAGA-3ʹ (forward) and 5ʹ-GGATCTCTAGCCAGGCACATTC-3ʹ (reverse); and *TUBB*, 5ʹ-CGTGTTCGGCCAGAGTGGTGC-3ʹ (forward) and 5ʹ-GGGTGAGGGCATGACGCTGAA-3ʹ (reverse). PCR cycling parameters were as follows: initial denaturation at 95 °C for 10 min, followed by 35 cycles at 95 °C for 10 s, at 60 °C for 10 s, and at 72 °C for 5 s. The expression levels of *PRL*, *IGFBP-1*, *PgR*, and *ERα* were determined using the 2^−ΔΔCt^ method with *TUBB* (β-tubulin) as the internal control [25, 26].

### Primary culture and evaluation of decidualization

Cancer-associated stromal cells were isolated from EC tissues using deoxyribonuclease I and collagenase. Next, cancer-associated stromal cells were cultured in a medium (DMEM; Gibco, Thermo Fisher Scientific) containing 4.5 g/L glucose, charcoal-filtered 10% FBS, and 1% antibiotic–antimycotic (Gibco, Thermo Fisher Scientific). After separation using a nylon cell strainer (pore size: 100 μm), the cancer-associated stromal cells were seeded at 12 × 10^5^ cells per well in 6-well plates with DMEM containing 10% FBS. Finally, the primary cultured cells were cultured in a medium containing MPA (10^–6^ M) + cAMP (0.5 mM) or metformin (1 mM) alone or in combination (MPA, cAMP, and metformin) for 8 days. The medium was replaced every 2 days. Total RNA was isolated from primary cultured EC-associated stromal cells and transcribed into cDNA, and the relative expression of *PRL*, *IGFBP-1*, *PgR*, and *ERα* was determined by comparison with the baseline expression. All supernatants of the medium on the eighth day were retrieved and centrifuged at 1500 × *g* for 5 min; the sample was then used to evaluate PRL level.

### Statistical analysis

Statistical analysis for the cell proliferation assay was performed using the Mann–Whitney *U* test. Comparisons between paired values were performed using the Wilcoxon signed-rank test. Differences between unpaired groups were analyzed using the Mann–Whitney *U* test. All comparisons were planned, and the tests were two-tailed. *P* < 0.05 was considered statistically significant. All statistical analyses were performed using SPSS software (version 23; IBM, Chicago, IL, USA).

## Results

### MPA for fertility-sparing treatment increased serum PRL levels via central and local mechanisms

**Fig. 1:**
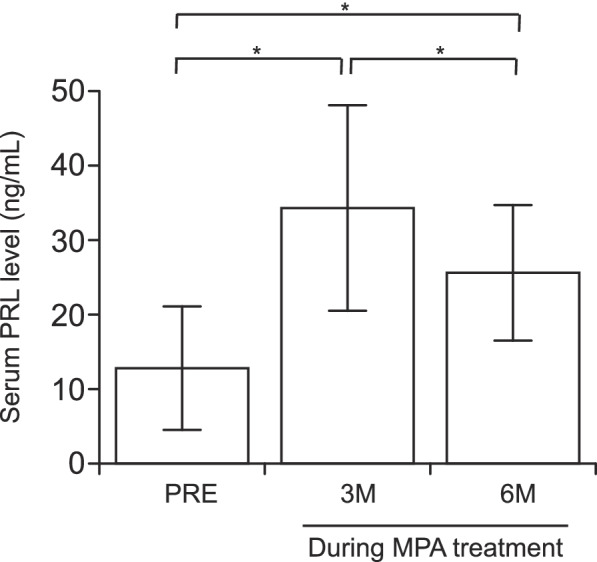
Serum prolactin levels during fertility-sparing treatment with MPA in patients with EC. Columns and error bars represent the mean ± standard deviation of the mean before treatment and after 3 and 6 months of MPA treatment. Wilcoxon signed-rank test was used and PRE indicates MPA pretreatment. (*P < 0.001). EC, endometrial cancer; MPA, medroxyprogesterone acetate

The mean serum PRL values (95% confidence interval) in patients with EAH and EC before treatment and at 3 months and 6 months after starting fertility-sparing treatment with MPA were 12.8 ng/mL (10.8–14.7 ng/mL), 34.7 ng/mL (31.5–37.9 ng/mL), and 25.2 ng/mL (23.2–27.3 ng/mL), respectively. Serum PRL levels during the fertility-sparing treatment increased significantly 3 months after starting MPA treatment (*P* < 0.001). The serum PRL value at 6 months was significantly lower than that at 3 months (*P* < 0.001) but remained higher than that before treatment (*P* < 0.001) (Fig. 1). Next, we compared the PRL levels between patients treated with MPA + metformin and those treated with MPA alone. No significant differences were observed in BMI and pathological type between the two groups. The mean serum PRL levels (95% confidence interval) in patients treated with MPA + metformin and MPA alone 3 months after starting fertility-sparing treatment were 12.8 ng/mL (10.8–14.7 ng/mL) and 25.2 ng/mL (23.2–27.3 ng/mL), respectively.

### Metformin could attenuate MPA-induced upregulation of PRL and IGFBP-1 mRNA expression during fertility-sparing treatment in patients with EC

IGFBP-1 is a marker of EC cell proliferation and metastasis and is involved in endometrial decidualization. Using samples from 16 patients with EC who were treated with MPA and treated with or without metformin, we examined the change in the mRNA expression levels of *PRL*, *IGFBP-1*, *PgR*, and *Erα* after MPA administration.

**Fig. 2:**
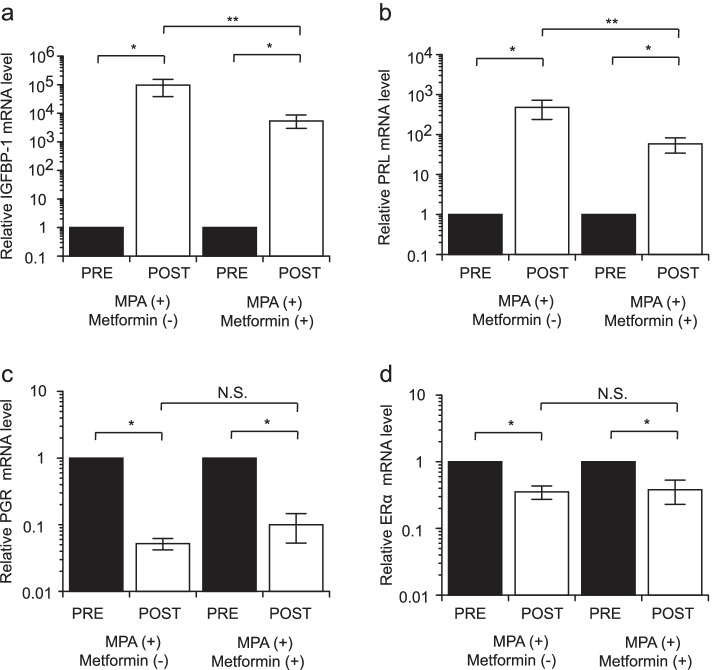
*IGFBP-1* **a**, *PRL* **b**, *PgR* **c**, and *ERα* **d** mRNA levels before and after MPA treatment. PRE: MPA pretreatment; POST: 3 months after MPA treatment; NS, not significant. (*P < 0.01, ** *P* < 0.05). ERα, estrogen receptor-α; IGFBP-1, insulin-like growth factor-binding protein 1; MPA, medroxyprogesterone acetate; PgR, progesterone receptor

MPA administration significantly elevated *IGFBP1* (mean proportional increase of 96,200-fold) and *PRL* (mean proportional increase of 479-fold) mRNA levels. In the case of MPA combined with metformin, the *IGFBP-1* and *PRL* mRNA levels were also significantly elevated, with a mean proportional increase of 5,370- and 58.4-fold, respectively. However, the addition of metformin significantly reduced the MPA-induced *IGFBP-1* and *PRL* mRNA expression to 18% (from 96,200-fold to 5,370-fold) and 8% (from 479-fold to 58.4-fold), respectively (Fig. 2a, b). These results suggest that metformin inhibits MPA-induced decidualization and PRL production during fertility-sparing treatment with MPA.

MPA administration resulted in a significant decrease in *PgR* and *ERα* mRNA levels (mean proportional decrease to 5.2% and 38.3%, respectively). However, the addition of metformin did not affect *PgR* and *ERα* mRNA expression (Fig. 2c, d).

### Metformin inhibited MPA-induced decidualization of cancer-associated stromal cells and production of PRL

To assess PRL production during MPA administration in fertility-sparing treatment, we performed an in vitro assay using primary culture of EC mesenchymal cells. We added MPA and cAMP to EC mesenchymal cells to establish a decidualization model.

**Fig. 3:**
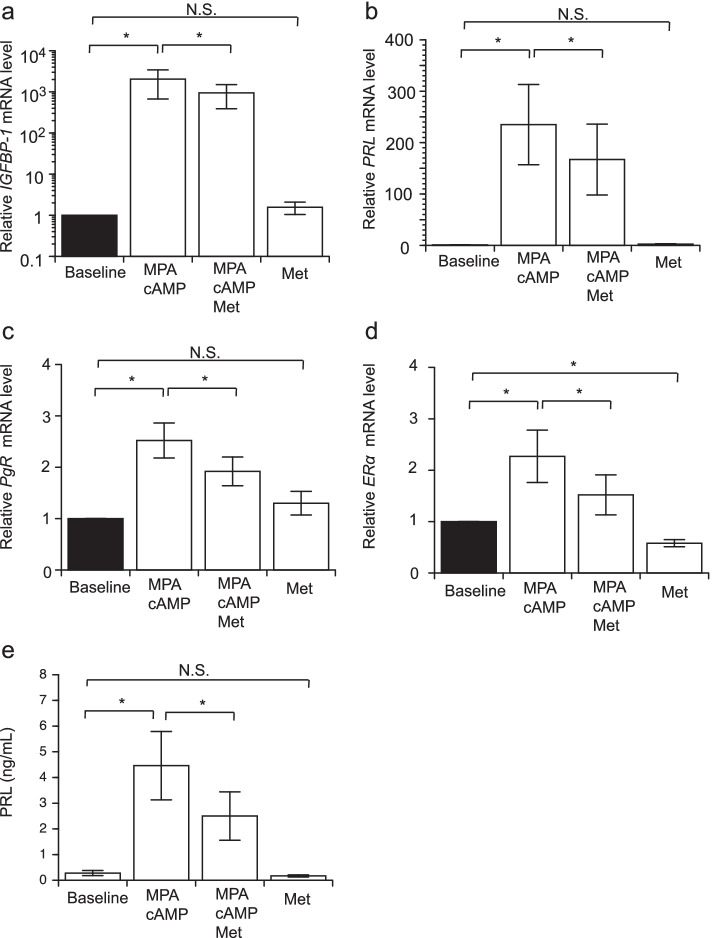
Decidualization of stromal cell by MPA and suppression by metformin. Stromal cell treatment with/without MPA (10^–6^ M), cAMP (0.5 mM), and with/without metformin (1 mM) (8 days). *IGFBP-1* **a**, *PRL* **b**, *PgR* **c**, and *ERα* **d** mRNA levels **e**. Columns, error bars represent the mean ± standard deviation of the mean. Met, metformin; NS, not significant (*P < 0.01, ** *P* < 0.05). MPA, medroxyprogesterone acetate

The addition of MPA and cAMP to the medium resulted in a significant increase in *IGFBP-1* and *PRL* mRNA expression compared with that in the control group (mean proportional increase of 2,048- and 235-fold, respectively). However, when metformin was added to the medium containing MPA and cAMP, the upregulation of *IGFBP-1* and *PRL* mRNA expression was significantly attenuated compared with that following the addition of MPA and cAMP without metformin (mean proportional decrease to 71% and 46%, respectively) (Fig. 3a, b).

*PgR* and *ERα* mRNA levels also increased after stimulation by MPA and cAMP. However, the addition of metformin significantly attenuated this increase compared with that in the absence of metformin (mean proportion decrease to 76% and 67%, respectively) (Fig. 3c, d).

Additionally, we cultured the cells with metformin for 8 days to evaluate the effects of metformin on PRL production. The amount of secreted PRL in the medium was elevated in EC mesenchymal cells 8 days after stimulation by MPA and cAMP. The mean PRL level in the culture supernatant containing MPA and cAMP was 4.1 ng/mL, which was 16.1-fold higher than that in the control (0.25 ng/mL). With the addition of metformin, the mean PRL level in the culture supernatant was 2.2 ng/mL. Thus, metformin significantly inhibited PRL secretion by decidualization induced by MPA and cAMP treatment (Fig. 3e).

### Metformin inhibited PRL-stimulated cancer cell proliferation in vitro

**Fig. 4:**
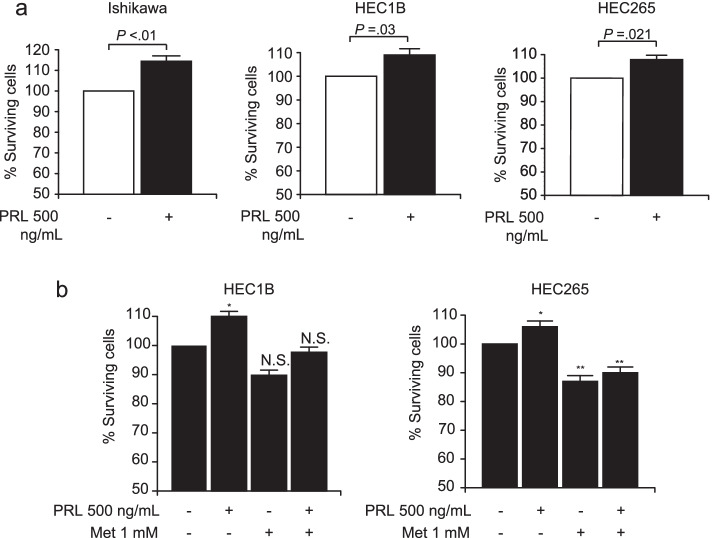
Viability of Ishikawa, HEC1B, and HEC265 cells exposed to 500 ng/mL PRL for various durations. Cell proliferation at 72 h after prolactin (PRL) treatment **a**. Effect of metformin on PRL-induced cell proliferation at 72 h **b**. Columns, error bars represent the mean ± standard deviation of the mean. (*P < 0.01, **P < 0.05)

We examined the effect of PRL on the proliferation of three EC cell lines (Ishikawa, HEC1B, and HEC265). PRL significantly stimulated growth in all EC cell lines at 72 h (*P* < 0.001 for Ishikawa, *P* = 0.03 for HEC1B, and *P* = 0.021 for HEC265; Fig. 4a).

Next, we examined the effects of metformin on the proliferative effects of PRL. Previously, we had observed an anti-proliferative effect of metformin on EC cell lines [27, 28]. The combination treatment of metformin, PRL, and metformin reduced the proliferative effects of PRL compared with those in cells treated with PRL alone (Fig. 4b).

### PRL induced the activation of ERK1/2 and metformin attenuated this activation

**Fig. 5:**
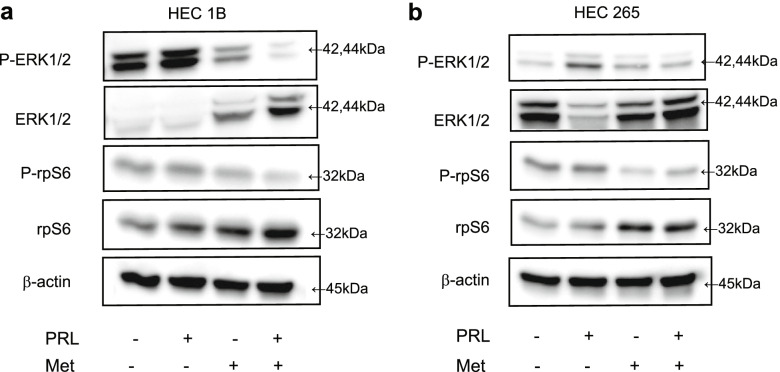
PRL induced the activation of ERK1/2 and rpS6 and metformin attenuated this effect. We preliminary observed that phospho-ERK1/2 levels were the most elevated at 6 h after the addition of PRL, and this increase was time-dependent (data not shown).Total protein extracted from HEC1B cells **a** and HEC265 cells **b** exposed to 500 ng/mL of PRL with or without 1 mM metformin for 6 h was subjected to western blotting. Met indicates metformin

The AMP-activated protein kinase (AMPK)/mechanistic target of rapamycin kinase (mTOR)/rpS6 and MAPK pathways are implicated in the proliferation of human EC. To confirm these findings, we examined the changes in phospho-ERK1/2 and phospho-rpS6 levels using western blotting. The addition of PRL increased phospho-ERK1/2 levels in both cell lines (by 22% and 250%); however, the phospho-rpS6 levels did not change (Fig. 5). Moreover, the addition of metformin reduced the PRL-induced increase in phospho-ERK1/2 levels by 93% and 70% (Fig. 5).

## Discussion

This study revealed the effects of PRL and metformin on the fertility-sparing treatment of EC using MPA. First, we demonstrated that serum PRL increased and *IGFBP-1* and *PRL* mRNA levels in EC tissues were upregulated during fertility-sparing treatment using MPA. Second, MPA might induce endometrial decidualization. This was suggested to be the cause of the increased serum PRL levels during fertility-sparing treatment using MPA. Third, we found that metformin suppressed the progesterone-induced decidualization of endometrial stromal cells and PRL production. Finally, we confirmed that PRL promoted the proliferation of EC cells, as shown previously [9]. We revealed, for the first time, that metformin attenuated the cell proliferation-promoting effect of PRL.

We confirmed that MPA administration increased serum PRL levels during fertility-sparing treatment. Progestin induces an increase in PRL levels via a central mechanism involving the dopamine axis [29]. Progestin has also been reported to induce decidualization of the endometrium and produce PRL [18]. Both mechanisms are thought to cause an increase in serum PRL levels during MPA therapy. Immediately after the start of treatment, we speculated that the PRL level increased because of decidualization of the endometrium, but as the treatment progressed, the endometrium became thinner and only the central mechanism of the increase in PRL levels remained. To date, there have been reports of decidualization in normal endometrial stromal cells by progestin with E2 or cAMP [30]. Metformin suppressed PRL production induced by decidualization of endometrial stromal cells. This was caused by a reduction in *IGFBP-1* gene expression after long-term metformin exposure [31]. In the current study, the administration of metformin suppressed decidualization and attenuated PRL production in a primary culture of EC stromal cells. In addition, even in the endometrium of patients undergoing fertility-sparing treatment, *PRL* and *IGFBP-1* mRNA levels were attenuated in the metformin-administered group and may have suppressed decidualization and PRL production.

We confirmed that PRL promoted cell proliferation and metformin attenuated this effect. *PRL* mRNA is expressed in ovarian and endometrial tumors, and exogenous PRL induces proliferation in several ovarian and EC cell lines via the Ras pathway [32]. EC cells transfected with the *PRL* gene have increased proliferative ability [9]. In vitro experiments have demonstrated that PRL increases cell proliferation through the activation of MAPK [33]. Previous reports have indicated that PRL rapidly increases phospho-ERK levels within 5 min [6, 8]; however, we demonstrated that the level of phospho-ERK increased after 6 to 12 h of addition of PRL. We confirmed that the early increase in phospho-ERK levels was due to the vigorous addition of PRL, and this early increase was avoided by the gentle addition of PRL (data not shown). Metformin inhibits cancer cell growth by activating AMPK and inhibiting the mTOR/ribosomal S6 kinase (S6K) and MAPK signaling pathways [34, 35]. The suppressive effect of metformin on PRL-induced cell proliferation might be mediated by the MAPK signaling pathways.

We speculate that PRL production during MPA administration might have a negative effect on fertility-sparing treatment. A study revealed that during fertility-sparing treatment, patients with serum PRL levels of 15 ng/mL or higher (mean serum PRL: 25.2 ng/mL) who are administered cabergoline have a better prognosis than patients who are not administered cabergoline because their serum PRL level is below 15 ng/mL (mean serum PRL: 12 ng/mL) [36]. This report suggests that maintaining a low serum PRL level during cabergoline administration improves prognosis. Thus, it can be inferred that increased PRL levels during fertility-sparing treatment adversely affect the treatment results. We previously reported that a combination of metformin and MPA during fertility-sparing treatment inhibits recurrence after remission [23, 24] via the direct effect of metformin and indirect effect of improved hormonal status [27]. In this study, we demonstrated that the inhibition of PRL production and its action may be involved in the recurrence-suppressing effect by metformin. In the future, the mechanism underlying the recurrence-suppressing effects of the combined use of metformin and MPA should be explored.

This study has some limitations. First, the extent of the in vivo adverse effects of PRL during actual conservative treatment remains unclear, as this study was an investigation of the effects on cell proliferation in vitro. Second, we could not reveal that metformin reduced serum PRL levels as compared to MPA alone in patients during fertility-sparing treatment. Moreover, there are selection biases between the two groups. And this study has small sample size and retrospective study. We believe that a prospective randomized study that verifies these findings is necessary. Third, we focused only on the local effect of metformin without investigating the central action of metformin in vivo. In this regard, several reports have revealed that adjunctive metformin significantly reduces PRL levels in patients with antipsychotic-induced hyperprolactinemia [37–39]. However, we could not evaluate this point. Further investigations are needed to reveal the clinical effects of metformin on PRL levels.

## Conclusions

Our study is the first to report an increase in PRL levels during fertility-sparing treatment using MPA. We showed that MPA administration could increase the production of PRL due to local decidualization in patients undergoing fertility-sparing treatment. Furthermore, we revealed that metformin antagonized the increase in local PRL production following MPA administration and attenuated the cell proliferation-promoting effect of PRL, which has positive effects on EC treatment outcomes. Although further research is needed on the underlying mechanisms, the findings of this study provide valuable insights on the application of metformin to improve the outcomes of fertility-sparing treatment.

## Supplementary Information

**Additional file 1:**

## Data Availability

The datasets generated during and analyzed during the current study are not publicly available due to confidentiality agreements but are available from the corresponding author on reasonable request.

## Abbreviations

AMPK: AMP-activated protein kinase
BMI: Body mass index
cAMP: Cyclic adenosine monophosphate
EAH: Endometrial atypical hyperplasia
EC: Endometrial cancer
ERα: Estrogen receptor-α
ERK: Extracellular signal-regulated kinase
HOMA-R: Homeostasis model assessment of insulin resistance
IGFBP: Insulin-like growth factor-binding protein
MAPK: Mitogen-activated protein kinase
mTOR: Mammalian target of rapamycin kinase
MPA: Medroxyprogesterone acetate
PCOS: Polycystic ovarian syndrome
PCR: Polymerase chain reaction
PgR: Progesterone receptor
PRL: Prolactin

## References

[1] Marano RJ, Ben-Jonathan N (2014). Minireview: Extrapituitary prolactin: An update on the distribution, regulation, and functions. Mol Endocrinol.

[2] Bao L, Tessier C, Prigent-Tessier A, Li F, Buzzio OL, Callegari EA (2007). Decidual prolactin silences the expression of genes detrimental to pregnancy. Endocrinology.

[3] Fernandez I, Touraine P, Goffin V (2010). Prolactin and human tumourogenesis. J Neuroendocrinol.

[4] Wu ZS, Yang K, Wan Y, Qian PX, Perry JK, Chiesa J (2011). Tumor expression of human growth hormone and human prolactin predict a worse survival outcome in patients with mammary or endometrial carcinoma. J Clin Endocrinol Metab.

[5] Stattin P, Rinaldi S, Stenman UH, Riboli E, Hallmans G, Bergh A (2001). Plasma prolactin and prostate cancer risk: A prospective study. Int J Cancer.

[6] Neradugomma NK, Subramaniam D, Tawfik OW, Goffin V, Kumar TR, Jensen RA (2014). Prolactin signaling enhances colon cancer stemness by modulating Notch signaling in a Jak2-STAT3/ERK manner. Carcinogenesis.

[7] Kong X, Wu W, Yuan Y, Pandey V, Wu Z, Lu X (2016). Human growth hormone and human prolactin function as autocrine/paracrine promoters of progression of hepatocellular carcinoma. Oncotarget.

[8] Domínguez-Cáceres MA, García-Martínez JM, Calcabrini A, González L, Porque PG, León J (2004). Prolactin induces c-Myc expression and cell survival through activation of Src/Akt pathway in lymphoid cells. Oncogene.

[9] Ding K, Yuan Y, Chong QY, Yang Y, Li R, Li X (2017). Autocrine prolactin stimulates endometrial carcinoma growth and metastasis and reduces sensitivity to chemotherapy. Endocrinology.

[10] Siegel RL, Miller KD, Fuchs HE, Jemal A (2022). Cancer statistics, 2022. CA Cancer J Clin.

[11] National Comprehensive Cancer Network. Uterine Cancer (Version 1.2022). https://www.nccn.org/professionals/physician_gls/pdf/uterine.pdf.Accessed Nov 4, 2021.

[12] Colombo N, Creutzberg C, Amant F, Bosse T, González-Martín A, Ledermann J, et al. ESMO-ESGO-ESTRO Consensus Conference on Endometrial Cancer: Diagnosis, treatment and follow-up. Ann Oncol. 2016;27:16–41. 10.1093/annonc/mdv484 26634381

[13] Gallos ID, Yap J, Rajkhowa M, Luesley DM, Coomarasamy A, Gupta JK (2012). Regression, relapse, and live birth rates with fertility-sparing therapy for endometrial cancer and atypical complex endometrial hyperplasia: A systematic review and metaanalysis. Am J Obstet Gynecol.

[14] Zhang Q, Qi G, Kanis MJ, Dong R, Cui B, Yang X (2017). Comparison among fertility-sparing therapies for well differentiated early-stage endometrial carcinoma and complex atypical hyperplasia. Oncotarget.

[15] Gunderson CC, Fader AN, Carson KA, Bristow RE (2012). Oncologic and reproductive outcomes with progestin therapy in women with endometrial hyperplasia and grade 1 adenocarcinoma: A systematic review. Gynecol Oncol.

[16] Chaudhury RR, Chompootaweep S, Dusitsin N, Friesen H, Tankeyoon M (1977). The release of prolactin by medroxy-progesterone acetate in human subjects. Br J Pharmacol.

[17] Chikosi AB, Parasztsak M, Ojwang P, Moodley J (1999). Prolactin levels in South African women on injectable progestogen contraceptives. S Afr Med J.

[18] Brosens JJ, Hayashi N, White JO (1999). Progesterone receptor regulates decidual prolactin expression in differentiating human endometrial stromal cells. Endocrinology.

[19] Decensi A, Puntoni M, Goodwin P, Cazzaniga M, Gennari A, Bonanni B (2010). Metformin and cancer risk in diabetic patients: A systematic review and meta-analysis. Cancer Prev Res (Phila).

[20] Evans JM, Donnelly LA, Emslie-Smith AM, Alessi DR, Morris AD (2005). Metformin and reduced risk of cancer in diabetic patients. BMJ.

[21] Ezewuiro O, Grushko TA, Kocherginsky M, Habis M, Hurteau JA, Mills KA (2016). Association of metformin use with outcomes in advanced endometrial cancer treated with chemotherapy. PLoS ONE.

[22] Nevadunsky NS, Van Arsdale A, Strickler HD, Moadel A, Kaur G, Frimer M (2014). Metformin use and endometrial cancer survival. Gynecol Oncol.

[23] Mitsuhashi A, Sato Y, Kiyokawa T, Koshizaka M, Hanaoka H, Shozu M (2016). Phase II study of medroxyprogesterone acetate plus metformin as a fertility-sparing treatment for atypical endometrial hyperplasia and endometrial cancer. Ann Oncol.

[24] Mitsuhashi A, Habu Y, Kobayashi T, Kawarai Y, Ishikawa H, Usui H (2019). Long-term outcomes of progestin plus metformin as a fertility-sparing treatment for atypical endometrial hyperplasia and endometrial cancer patients. J Gynecol Oncol.

[25] Pfaffl MW (2001). A new mathematical model for relative quantification in real-time RT-PCR. Nucleic Acids Res.

[26] Livak KJ, Schmittgen TD (2001). Analysis of relative gene expression data using real-time quantitative PCR and the 2(-delta delta C(T)) method. Methods.

[27] Mitsuhashi A, Kiyokawa T, Sato Y, Shozu M (2014). Effects of metformin on endometrial cancer cell growth in vivo: A preoperative prospective trial. Cancer.

[28] Uehara T, Mitsuhashi A, Tsuruoka N, Shozu M (2015). Metformin potentiates the anticancer effects of cisplatin under normoxic conditions in vitro. Oncol Rep.

[29] Molitch ME (2005). Medication-induced hyperprolactinemia. Mayo Clin Proc.

[30] Xiong F, Xiao J, Bai Y, Zhang Y, Li Q, Lishuang X (2019). Metformin inhibits estradiol and progesterone-induced decidualization of endometrial stromal cells by regulating expression of progesterone receptor, cytokines and matrix metalloproteinases. Biomed Pharmacother.

[31] Germeyer A, Jauckus J, Zorn M, Toth B, Capp E, Strowitzki T (2011). Metformin modulates IL-8, IL-1beta, ICAM and IGFBP-1 expression in human endometrial stromal cells. Reprod Biomed Online.

[32] Levina VV, Nolen B, Su YY, Godwin AK, Fishman D, Liu J (2009). Biological significance of prolactin in gynecologic cancers. Cancer Res.

[33] Karthikeyan S, Russo A, Dean M, Lantvit DD, Endsley M, Burdette JE (2018). Prolactin signaling drives tumorigenesis in human high grade serous ovarian cancer cells and in a spontaneous fallopian tube derived model. Cancer Lett.

[34] Quinn BJ, Kitagawa H, Memmott RM, Gills JJ, Dennis PA (2013). Repositioning metformin for cancer prevention and treatment. Trends Endocrinol Metab.

[35] Pernicova I, Korbonits M (2014). Metformin-Mode of action and clinical implications for diabetes and cancer. Nat Rev Endocrinol.

[36] Erdenebaatar C, Yamaguchi M, Saito F, Monsur M, Honda R, Tashiro H (2018). Administration of cabergoline contributes to preserving fertility in young hyperprolactinemic patients with endometrial cancer treated with medroxyprogesterone acetate. Int J Gynecol Cancer.

[37] Krysiak R, Kowalcze K, Szkrobka W, Okopien B (2016). The effect of metformin on prolactin levels in patients with drug-induced hyperprolactinemia. Eur J Intern Med.

[38] Bo QJ, Wang ZM, Li XB, Ma X, Wang CY, de Leon J (2016). Adjunctive metformin for antipsychotic-induced hyperprolactinemia: A systematic review. Psychiatry Res.

[39] Zheng W, Yang XH, Cai DB, Ungvari GS, Ng CH, Wang N (2017). Adjunctive metformin for antipsychotic-related hyperprolactinemia: A meta-analysis of randomized controlled trials. J Psychopharmacol.

