# Supplementary material for: Metformin attenuates the production and proliferative effects of prolactin induced by medroxyprogesterone acetate during fertility-sparing treatment for endometrial cancer
Source: BMC Cancer. 2022 Jul 11;22:753. doi: 10.1186/s12885-022-09858-w (PMC9277913; doi:10.1186/s12885-022-09858-w)

Supplementary file 1

Supplementary file 1 shows the original image of Figure 5 before cropping.
Contrast was not adjusted using any image processing software.
Dr.Western (ORIENTAL YEAST , Japan)was used as molecular marker.

Fig 5 a HEC1B cell

P-ERK 44, 42kDa

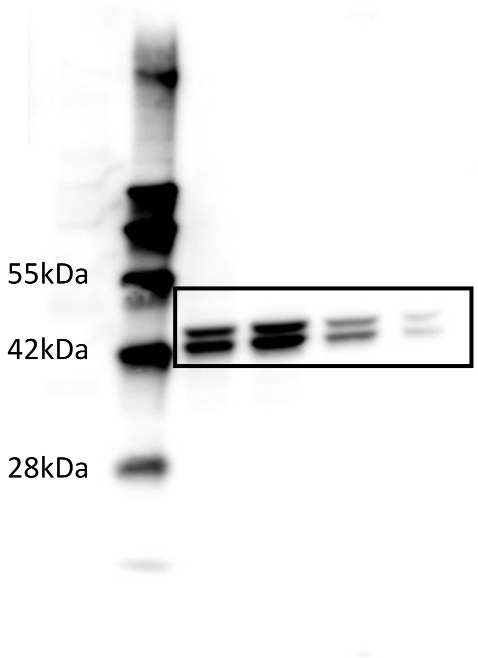

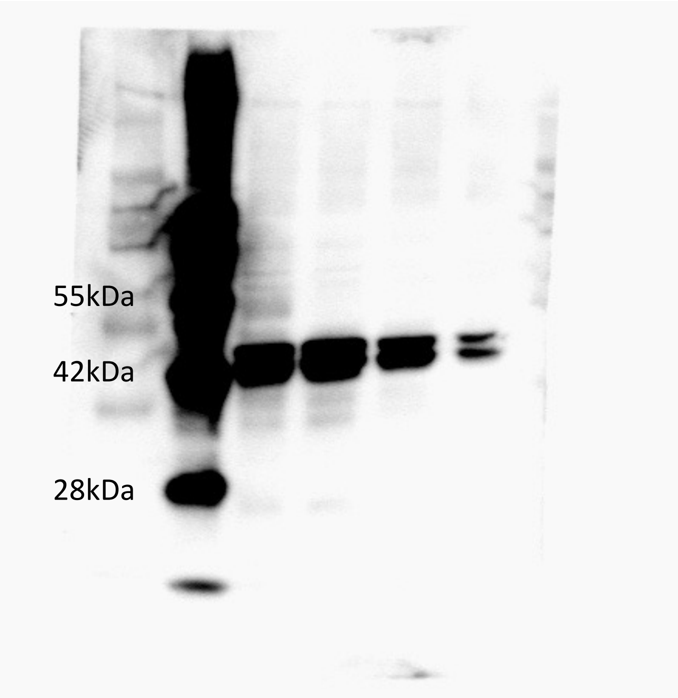

ERK 44, 42kDa

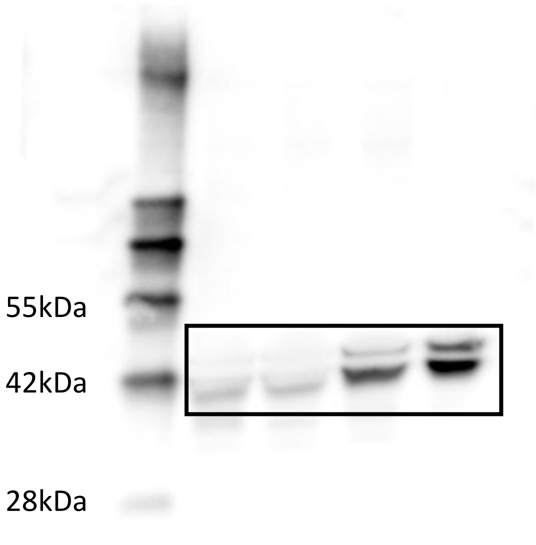

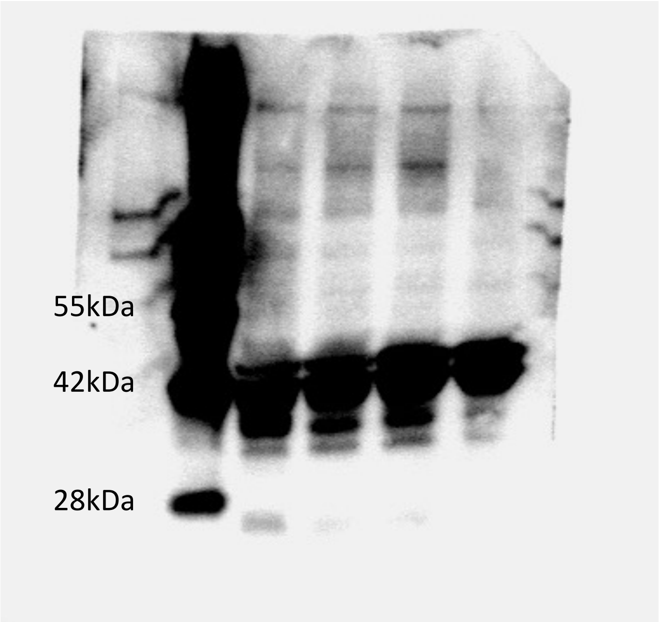

P-rpS6 32kDa

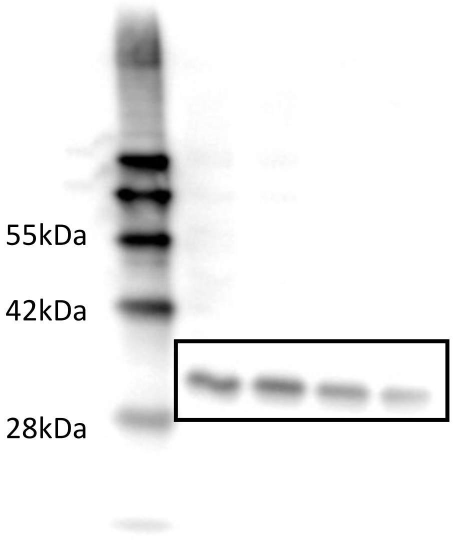

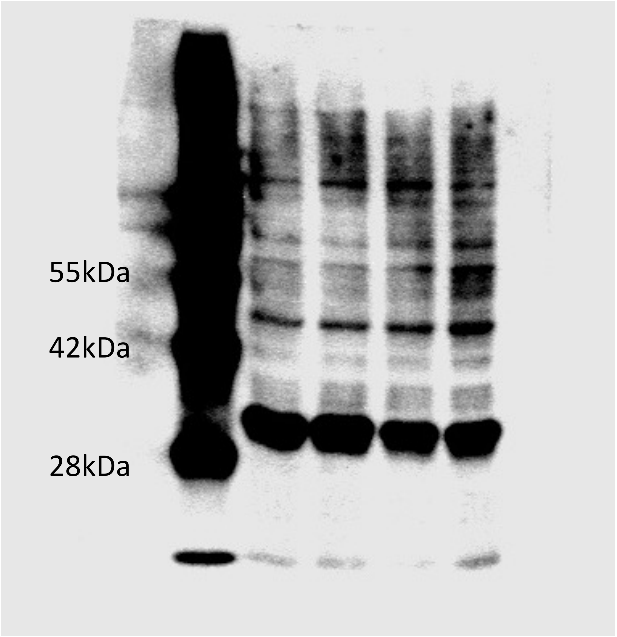

rpS6 32kDa

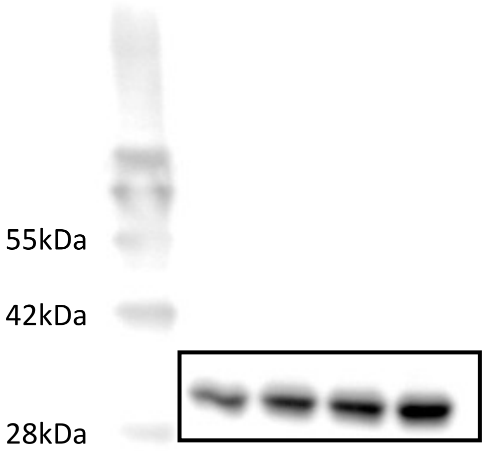

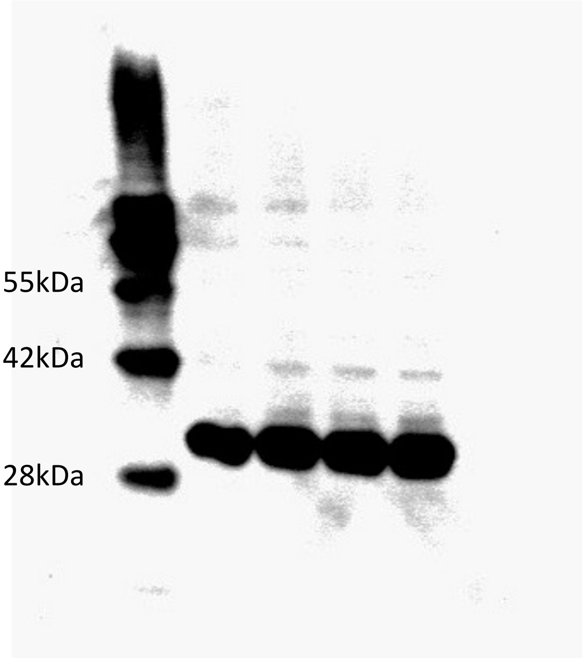

β actin 45kDa

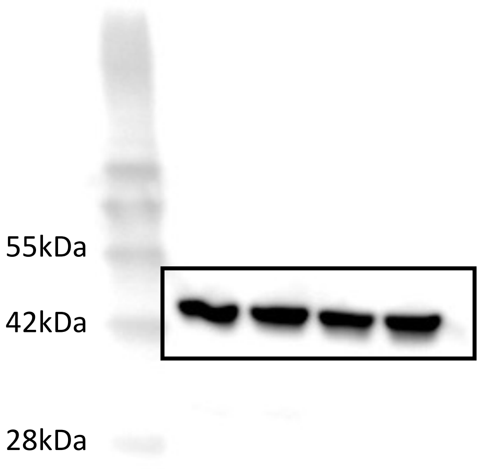

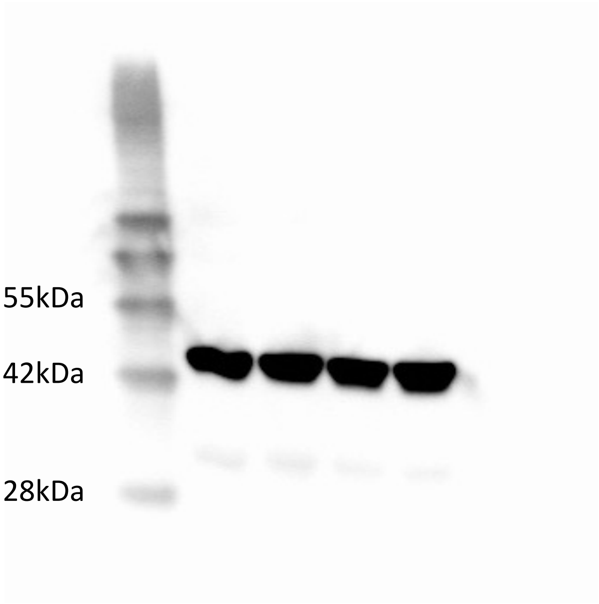

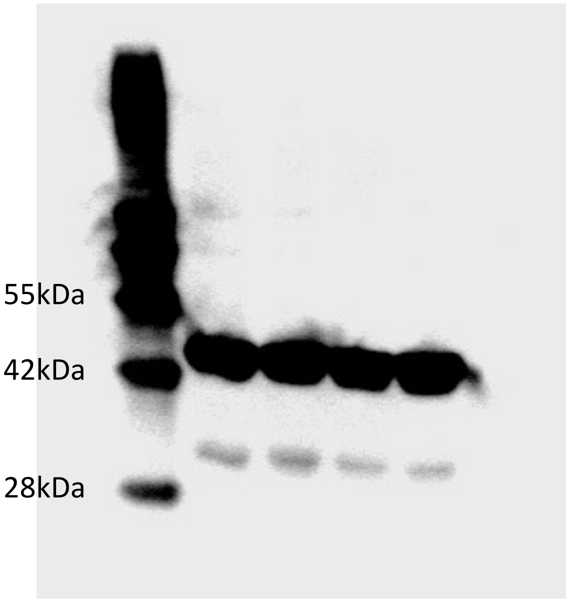

Fig 5 b HEC 265cell

P-ERK 44, 42kDa

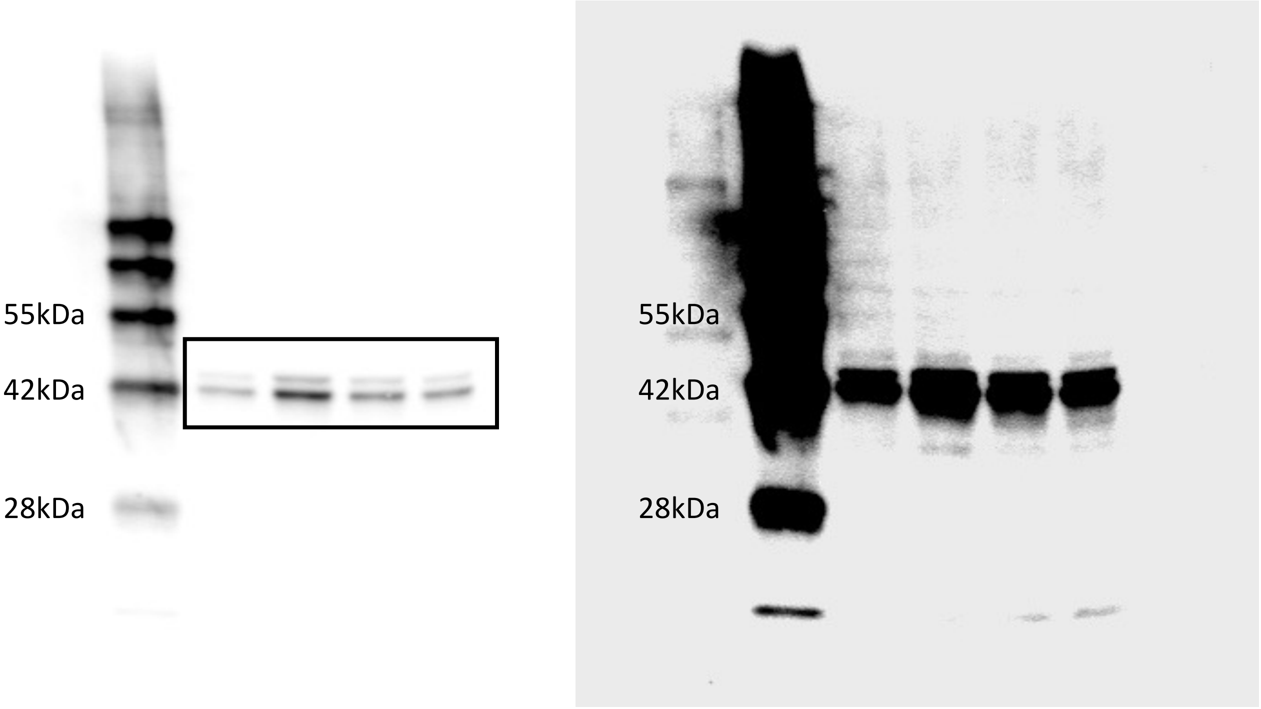

ERK 44, 42kDa

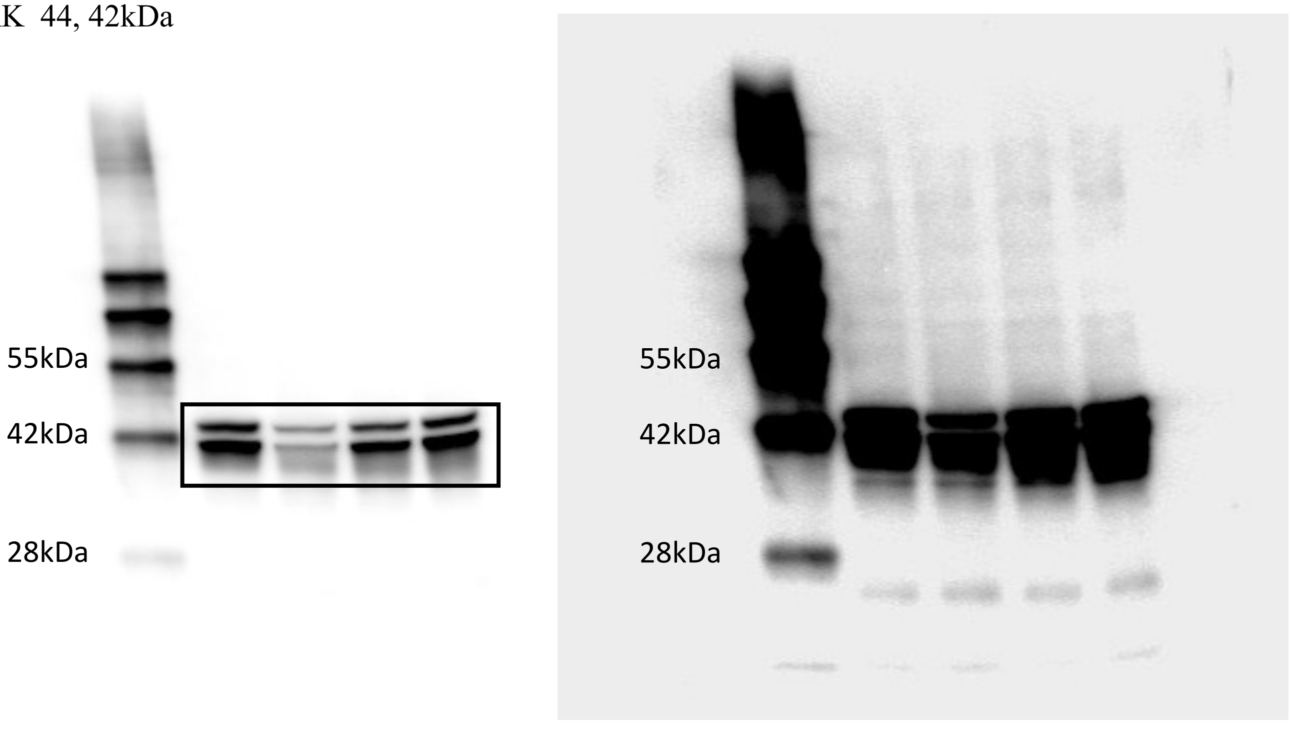

P-rpS6 32kDa

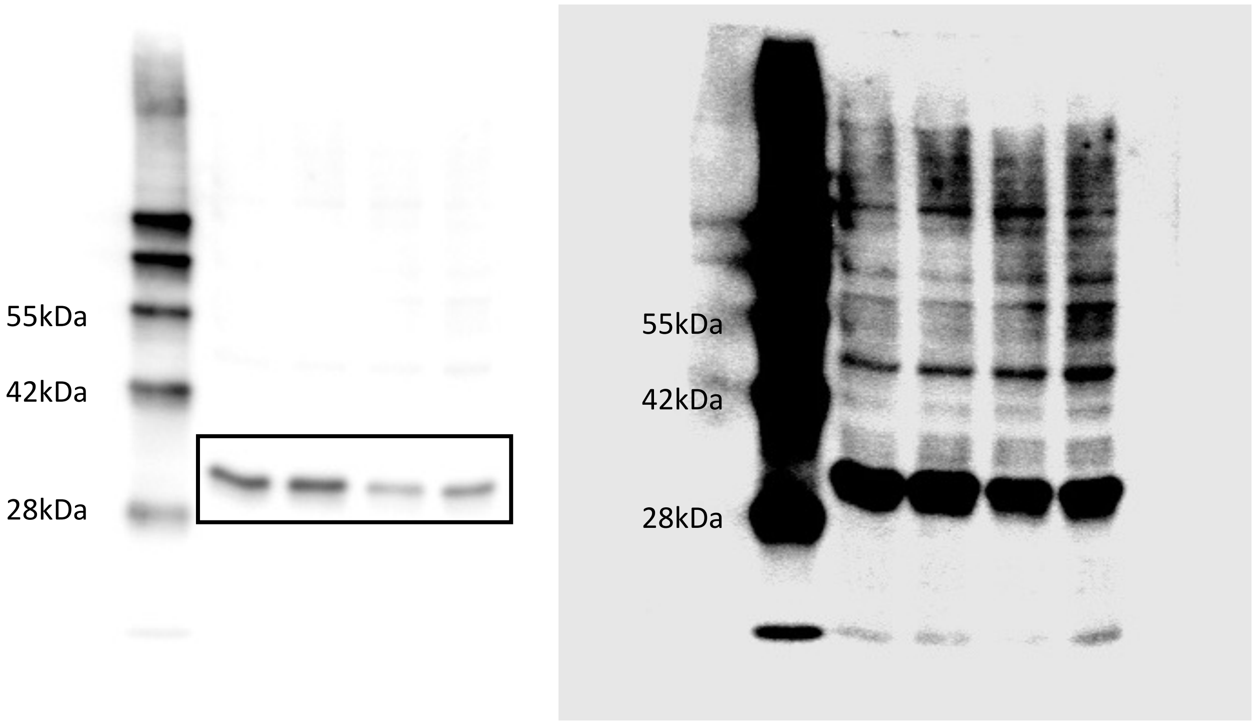

rpS6 32kDa

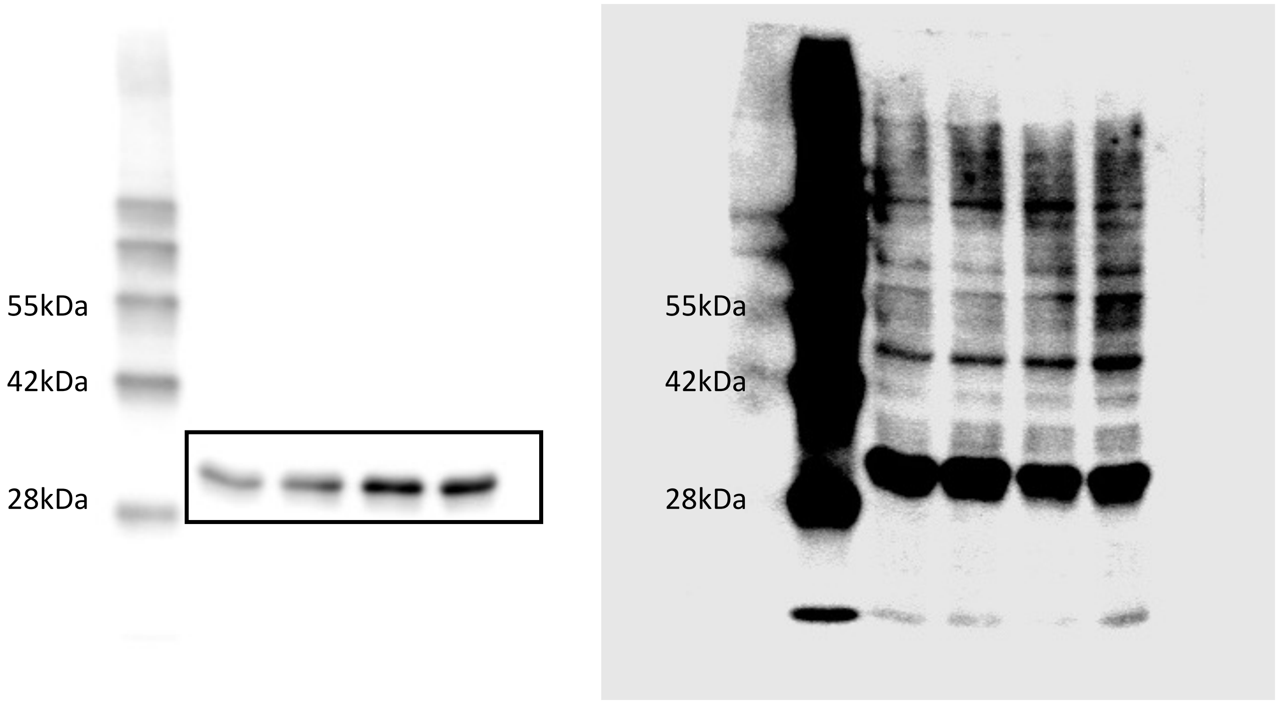

β actin 45kDa

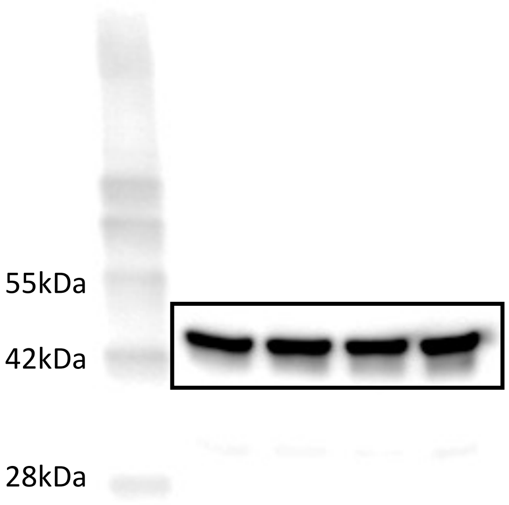

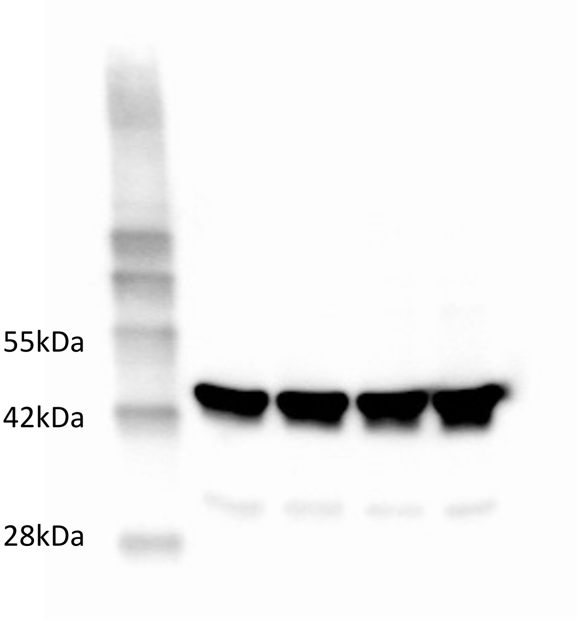

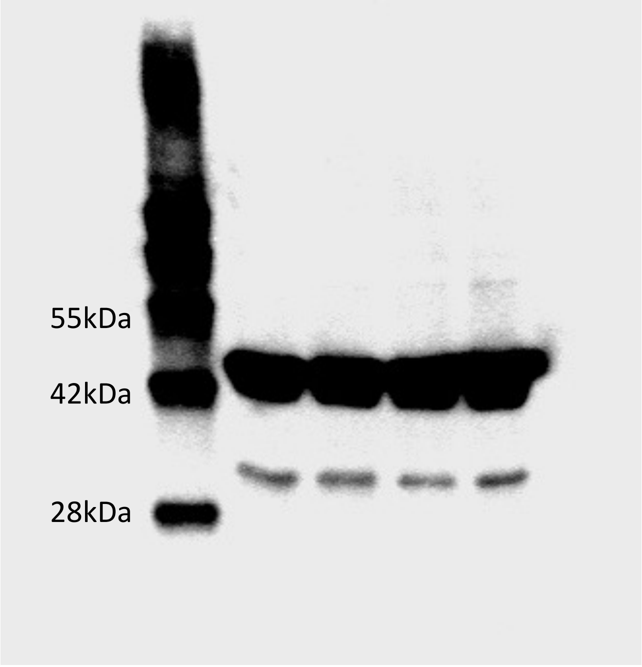

Supplement: Supplementary file 1 — Additional file 1: [file 12885_2022_9858_MOESM1_ESM.pdf]
